# Effects of Fat and Sugar, Either Consumed or Infused toward the Brain, on Hypothalamic ER Stress Markers

**DOI:** 10.3389/fnins.2017.00270

**Published:** 2017-05-15

**Authors:** Evita Belegri, Merel Rijnsburger, Leslie Eggels, Unga Unmehopa, Wiep Scheper, Anita Boelen, Susanne E. la Fleur

**Affiliations:** ^1^Laboratory of Endocrinology, Department of Clinical Chemistry and Department of Endocrinology and Metabolism, Academic Medical Center, University of AmsterdamAmsterdam, Netherlands; ^2^Clincal Genetics, VU Medical CenterAmsterdam, Netherlands; ^3^Metabolism and Reward Group, Netherlands Institute for NeuroscienceAmsterdam, Netherlands

**Keywords:** ER stress response, hypothalamus, food restriction, fatty acids, sugar

## Abstract

Protein-folding stress at the Endoplasmic Reticulum (ER) occurs in the hypothalamus during diet-induced obesity (DIO) and is linked to metabolic disease development. ER stress is buffered by the activation of the unfolded protein response (UPR), a controlled network of pathways inducing a set of genes that recovers ER function. However, it is unclear whether hypothalamic ER stress during DIO results from obesity related changes or from direct nutrient effects in the brain. We here investigated mRNA expression of UPR markers in the hypothalamus of rats that were exposed to a free choice high-fat high-sugar (fcHFHS) diet for 1 week and then overnight fed *ad libitum*, or fasted, or fat/sugar deprived (i.e., switched from obesogenic diet to chow). In addition, we determined the direct effects of fat/sugar on mRNA expression of hypothalamus UPR markers by intracarotic infusions of intralipids and/or glucose in chow-fed rats that were fasted overnight. Short term (1 week) exposure to fcHFHS diet increased adiposity compared to chow-feeding. Short term exposure to a fcHFHS diet, followed by mild food restriction overnight, induced hypothalamic ER stress in rats as characterized by an increase in spliced to unspliced X-box binding protein 1 mRNA ratio in hypothalamus of fcHFHS fed rats compared to chow fed rats. Moreover, infused lipids toward the brain of overnight fasted rats, were able to induce a similar response. Non-restricted *ad libitum* fcHFHS-diet fed or totally fasted rats did not show altered ratios. We also observed a clear increase in hypothalamic activating transcription factor 4 mRNA in rats on the fcHFHS diet while being *ad libitum* fed or when infused with intralipid via the carotic artery compared to vehicle infusions. However, we did not observe induction of downstream targets implying that this effect is a more general stress response and not related to ER stress. Overall, we conclude that the hypothalamic stress response might be a sensitive sensor of fat and energy status.

## Introduction

Obesity is the result of a mismatch between energy intake and energy expenditure. Although a sedentary lifestyle can contribute to obesity development, the consumption of sugar-sweetened beverages and high amounts of saturated fat in foods has been linked to the risk to become obese and develop metabolic disorders (Hall et al., 2012). Over the last decades, the intake of sugar-based beverages has clearly increased worldwide, and from recent surveys it has become clear that consumption of both sugar-sweetened beverages and saturated fats (especially from snack foods) exceeds recommended daily levels (Popkin et al., 2012). It is, therefore, of utmost importance to understand how fat and sugar affect energy balance.

The hypothalamus is an important brain area in regulating energy balance (Elmquist and Flier, 2004; Schwartz and Porte, 2005). Under normal conditions the hypothalamus senses whole body energy demands via nutrient, neuronal and hormonal signaling and adjusts feeding behavior and energy expenditure via the production of orexigenic and anorexigenic peptides. For example leptin, the hormone secreted by adipose tissue in proportion to fat mass, activates the release of α-melanocyte stimulating hormone [α-MSH; derived from pro-opiomelanocortin (POMC)] and inhibits the release of the orexigenic neuropeptide Y (NPY) and agouti related peptide (AgRP) (Sahu, 2011). During obesity, however, the function of intracellular organelles in AGRP/NPY or POMC neurons like the endoplasmic reticulum (ER) was shown to be impaired leading to disturbed leptin signaling and energy imbalance (Hosoi et al., 2008; Zhang et al., 2008; Ozcan et al., 2009; Cakir et al., 2013; Ramírez and Claret, 2015).

The ER is a complex membrane network responsible for the synthesis and folding of various transmembrane and secreted proteins (Westrate et al., 2015). Accumulation of toxic, misfolded proteins in the ER leads to ER stress and activation of the unfolded protein response (UPR). The UPR is a network of pathways controlled by three sensors; PKR-like ER kinase (PERK), the activating transcription factor 6 (ATF6) and the inositol-requiring protein-1 alpha (IREα). Activation of these pathways induces the expression of genes that lead to the expansion of the ER, reduction of protein translation and increase of protein folding capacity promoting cell survival or induces apoptosis (Walter and Ron, 2011; Lee and Ozcan, 2014). Upon PERK activation, activating transcription factor 4 (ATF4) mRNA is translated which increases the transcription of specific UPR target genes, including C/EBP homologous protein (CHOP) (Harding et al., 2000; Han et al., 2013). Immunoglobulin-heavy-chain-binding protein (BiP) mRNA is increased upon ATF6 activation and splicing of unspliced X box binding protein 1 to spliced XBP1 (sXBP1/usXBP1) occurs upon IREα activation (Yoshida et al., 2001). Both usXBP1 and sXBP1 were related to cell viability under ER stress conditions. Under severe ER stress, IREα activation can also lead to degradation of mRNAs and miRNAs and cell apoptosis via the c-Jun N-terminal kinase (JNK) pathway (Todd et al., 2008). DP5 (Death protein 5/harakiri) and FasL (Fas ligand) are genes expressed downstream of JNK indicating activation of the IREα—apoptotic pathway (Schenkel, 2004; Guan et al., 2006; Ma et al., 2007).

Long term high fat diet (HFD) feeding, resulting in profound body weight gain, induces hypothalamic ER stress characterized by increased protein levels of UPR markers like p-PERK, p-IREα, and phospho-eukaryotic initiation factor 2 alpha (p-eIF2α) (Ozcan et al., 2009; Cakir et al., 2013). Activation of UPR pathways was also reported when fatty acids (FA) [arachidic acid, palmitic acid, or ceramide] were directly supplied to the brain via intracerebroventricular (ICV) infusion in rodents (Milanski et al., 2009; Contreras et al., 2014) or when administered to murine neuronal cell lines (Choi et al., 2010) implying a more direct role for FA in hypothalamic ER stress induction. Although many of the HFD used also contain considerable amounts of sugar, the effects of sugar alone on the ER stress induction has not been studied so far.

The fact that long term HFD feeding, but also ICV infusions of FA, induce hypothalamic ER stress marker expression points to the idea that nutrients like FA can induce hypothalamic ER stress. However, it is not clear whether other nutrients or the increased adiposity reflecting a positive energy status or a combination of nutrients and energy status affects hypothalamic ER stress. We therefore determined whether overfeeding/fasting status and its interaction with diet-induced obesity affected genetic markers for ER stress in the hypothalamus. We exposed male Wistar rats to a free choice high-fat high-sugar (fcHFHS) diet or chow for 1 week followed by overnight *ad libitum* feeding, fat/sugar deprivation or fasting. In addition, we investigated if fat and sugar have a direct effect on the ER stress markers in the hypothalamus by intracarotic infusions of Intralipids (IL), IL and glucose, or glucose to the brain of overnight fasted lean rats. For both experiments, mRNA of different UPR markers was measured using RT-PCR as an indication for the induction of hypothalamic ER stress.

## Materials and methods

### Animals

Adult male Wistar rats (250–280 g, Charles River, Germany) were individually housed in a temperature controlled room (19 ± 1°C) on a 12 h light/ 12 h dark cycle (lights on at 7:00 a.m.). During the experiments animals had *ad libitum* access to water and standard laboratory chow (SDS, UK) unless stated differently. All the studies were approved by and performed according to the regulations of the Committee for Animal Experimentation of the Academic Medical Centre of the University of Amsterdam, Netherlands.

### fcHFHS diet experiments

Three experiments were performed to study the effect of nutrient availability on the hypothalamic ER stress response and its interaction with obesity. In all experiments rats were on a fcHFHS diet or chow for 1 week. The fcHFHS diet consisted of *ad libitum* access to chow, tap water, 30% sugar water (1.0 M sucrose mixed from commercial grade sugar and water) and a dish of pure animal fat (beef tallow; Ossewit/Blanc de Boeuf, Vandermoortele, Belgium) (composition: 34% oleic acid, 25% plamitc, and 22% stearic acid and 4% linoleic acid). After this period the three experiments differed in feeding regime the night before the end of the experiment (4:00 p.m. day 7–9:00 a.m. day 8).

- *experiment 1*: rats had *ad libitum* access to the control chow diet or to the fcHFHS diet.
- *experiment 2*: all rats received 10 g chow overnight (fat/sugar deprivation for rats on fcHFHS).
- *experiment 3*: all rats were fasted overnight.

Fat/sugar deprivation in our diet model is characterized by removing the fat/sugar components of the diet (i.e., saturated fat and sugar water) overnight. We previously showed that removing fat and sugar from the fcHFHS diet results in consumption of 10–15 g chow spontaneously without caloric compensation for the fat/sugar components of the diet (Pandit, 2015). To ensure equal intake overnight between fcHFHS and chow-fed rats we provided all animals with 10 g of chow. Food components of the diet were weighed 5 times a week and the amounts of components eaten were multiplied with the caloric value of each component to determine energy intake in kcals.

At the end of the experiment, rats were anesthetized via 30% CO_2_/70% O_2_ and decapitated between 9:00 a.m. and 11:00 a.m. Epididymal, mesenteric, subcutaneous and peritoneal fat were dissected and weighed, trunk blood collected and brains were removed and stored at −80°C until further analysis. Plasma leptin concentrations were determined by radioimmunoassay (Linco Research, Inc., St. Charles, MO, USA). Samples were assayed in duplicate. Amounts of sample, standards, label, antibody and precipitating reagent as described in the procedures of the assay were divided by 4. The detection limit was 0.5 ng/ml and the inter- and intra-assay coefficients were 8% or less.

### Intracarotic infusion experiment

Rats (*n* = 6–9 per group) underwent surgery under anesthesia induced with an i.p. injection of 80 mg/kg Ketamin (Eurovet Animal Health, Netherlands), 8 mg/kg Xylazin (Bayer Health Care) and 0.1 mg/kg Atropin (Pharmachemie, Netherlands). A silicon catheter was inserted in the carotid artery and directed toward the brain (according to the method of Steffens, 1969). The catheter was externalized at the vertex of the head and the animals were allowed to recover for 7 days.

To study the direct effect of fat and/or glucose on hypothalamic ER stress markers, NaCl (control), 20% IL [(Fresenuis Kabi); composition of IL is displayed in Table 1] or IL + 1% glucose (G) were infused via the carotid artery toward the brain (*experiment 4*). Another infusion experiment was performed to study the effect of glucose on hypothalamic ER stress response using glucose (1% in NaCl) or NaCl (*experiment 5*). All solutions were heparinized (0.04%) and Infusion rate was 5 μl/min for 2 h. One hour after the end of infusion the animals received a single shot of pentobarbital via the carotid artery (100–150 mg/kg BW) and were decapitated. Brains were removed and stored at −80°C for further analysis.

**Table 1:** **Content of intralipid 20%**.

**Fatty acid**	**Amount (%)**
Linoleic acid	52
Oleic acid	22
Palmitic acid	13
Linolenic acid	8
Stearic acid	4
Myristic acid	<1
Others	1

*Source; Fresenius Kabi*.

### Brain harvesting—isolation of the hypothalamus

Coronal brain slices of 250 μm were obtained from −0.96 to −4.36 mm Bregma (Rat brain atlas; Paxinos and Watson, 2007) and directly put in RNAlater solution (Ambion Life Technologies). The hypothalamic part in each section was isolated using syringe needles (0.4 × 19 mm, BD Microlance) and used for RT-PCR.

### RNA isolation—RT-PCR

One half of the hypothalamus was homogenized in lysis buffer provided with the “High Pure RNA isolation kit” (Roche Molecular Biochemicals, Manheim, Germany) and total RNA was isolated according to the manufacturer's instructions. RNA was quantified by spectrophotometry at 260 nm (Nanodrop 1000, Willmington, Delaware, USA) and cDNA synthesis was performed using the “Transcriptor First Strand cDNA synthesis kit” for RT PCR with oligo(dT) primers (Roche Molecular Biochemicals, Manheim, Germany). The mRNA levels of ER stress markers as well as Hypoxanthine-guanine phosphoribosyltransferase (Hprt), Cyclophilin A and β-actin were determined by RT-PCR using SensiFAST SYBR No-Rox mix (Bioline, Luckenwalde, Germany) at the Lightcycler 480 apparatus (Roche Molecular Biochemicals, Manheim, Germany). The primers were designed using “Primer Blast” (Table 2). Samples were baseline corrected and individually checked for their PCR efficiency using the “LC480 Conversion” and “LinRegPCR” software. Median efficiency was calculated for each assay and samples that differed more than 0.05 from the mean efficiency were excluded from statistical analysis. Specific gene expression was normalized to the geometric mean of three housekeeping genes; (Hprt × β-actin × Cyclophilin A)^1/3^.

**Table 2:** **Primer sequences used for RT-PCR**.

**Primers**	**Forward 5′–3′**	**Reverse 5′–3′**
ATF4	CTGAACAGCGAAGTGTTGGC	TCTGTCCCGGAAAAGGCATC
us-XBP1	GTCCGCAGCACTCAGACTAC	ATGAGGTCCCCACTGACAGA
s-XBP1	CTGAGTCCGAATCAGGTGCAG	ATCCATGGGAAGATGTTCTGG
DP5	ATGAAGCTGTGTTGCCGAGA	GCTTCAGTCCCACAGACTCC
FasL	TATCCTGGGGATCTGGTGCTA	TGCAGGCATTAAGGACCACT
Ddit3 (CHOP)	AGAGTGGTCAGTGCGCAGC	CTCATTCTCCTGCTCCTTCTCG^*^
Hspa (Bip)	TGGGTACATTTGATCTGACTGGA	CTCAAAGGTGACTTCAATCTGGG^*^
β-actin	CATGTACGTAGCCATCCAGGC	CTCTTTAATGTCACGCACGAT
Cyclophilin	ATGTGGTCTTTGGGAAGGTG	GAAGGAATGGTTTGATGGGT
HPRT	GCAGTACAGCCCCAAAATGG	AACAAAGTCTGGCCTGTATCCAA

* *Oslowski and Urano (2011)*.

### Statistical analysis

Data are presented as Mean ± SEM. Outliers were detected using “Dixon's *Q* test” and excluded. Differences between diet groups were evaluated using *Student's t-test* [experiments 1–4, 5 (NaCl vs. G)] or ANOVA followed by *post-hoc* Tukey test (experiment 5, NaCl vs. IL vs. IL+G). Difference between groups was considered significant when *p* < 0.05. In order to determine the effects of low vs. high fat intake and low vs. high sugar intake a median split was performed whereby the median was calculated for fat or for sugar intake and the animals that consumed more than the median were depicted as high consumers and those that consumed lower as low consumers. All tests were performed using Graphpad Prism 6 (Graphpad software Inc., la Jolla, CA, USA).

## Results

### One week of fcHFHS diet does not induce hypothalamic ER stress markers

Rats exposed to the fcHFHS diet for 1 week were hyperphagic as shown by increased caloric intake compared to those on chow (Table 3). As a result, % WAT/BW and plasma leptin levels were significantly higher in rats on the fcHFHS diet compared to rats on chow whereas ΔBW did not differ between the groups (Table 3).

**Table 3:** **Characteristics of chow and fcHFHS animals**.

**Exp**.	**ΔBW (gr)**		**% WAT/BW**		**Leptin (ng/ml)**		**Average intake/day (Kcal)**	
	**Chow**	**fcHFHS**	**Chow**	**fcHFHS**	**Chow**	**fcHFHS**	**Chow**	**fcHFHS**
1	34 ± 2.2	37 ± 2.3	2.58 ± 0.09	3.41 ± 0.05^****^	4.9 ± 0.8	7.6 ± 0.6^**^	76.0 ± 1.5	100 ± 2.5^***^
2	41 ± 3.4	52 ± 5.1	1.94 ± 0.13	2.81 ± 0.12^***^	2.01 ± 0.2	2.73 ± 0.3^***^	81.6 ± 4.4	122.1 ± 3.7^****^
3	51 ± 4.8	54 ± 3.7	2.20 ± 0.16	2.72 ± 0.16^*^	2.34 ± 0.3	3.24 ± 0.4^**^	88.0 ± 3.8	121.2 ± 5.5^***^

Delta body weight, average food intake per day, % total white adipose tissue (WAT) relative to final body weight of animals and leptin plasma concentrations in experiment 1 (ad lib fed o/n), 2 (10 g chow fed o/n) and experiment 3 (fasted o/n) are shown as mean (n = 8) ± SEM. Total WAT represents the sum of mesenteric, peritoneal, subcutaneous and epididymal fat. Significant differences between the fcHFHS and chow control group:

* *p < 0.05*,

** *p < 0.01*,

*** *p < 0.001*,

**** *p < 0.0001*.

**Figure 1:**
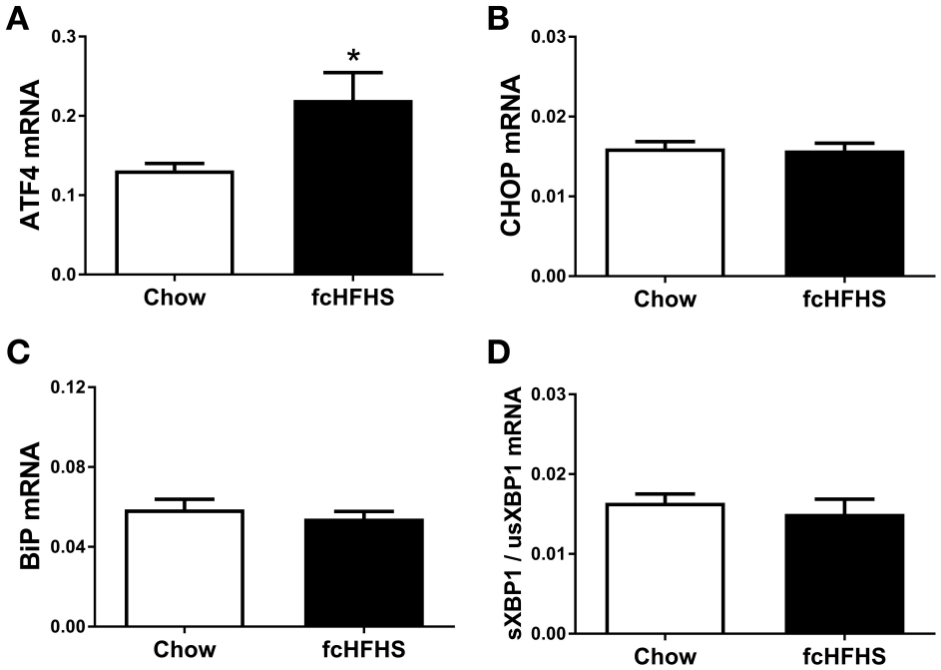
**ER stress markers in rat hypothalamus after 1 week ***ad libitum*** fcHFHS diet or chow. (A)** ATF4 and **(B)** CHOP **(C)** BiP, and **(D)** sXBP1/usXBP1 mRNA expression. mRNA expression of specific genes was normalized to the geometric mean of three housekeeping genes. Significant differences between the fcHFHS and control group: ^*^p < 0.05.

One week of fcHFHS diet exposure significantly increased ATF4 mRNA expression (Figure 1A). However, mRNA expression of CHOP, a target gene of ATF4, and BiP, a target gene of ATF6, was not significantly changed in hypothalami of fcHFHS-fed rats compared to chow-fed controls (Figures 1B,C). Splicing of usXBP1 to sXBP1 as shown by the ratio sXBP1/usXBP1 (Figure 1D), as well as DP5 and FasL mRNA expression did not differ between the groups.

### Overnight fat/sugar deprivation induces ER stress in animals exposed to the fcHFHS diet for 1 week

**Figure 2:**
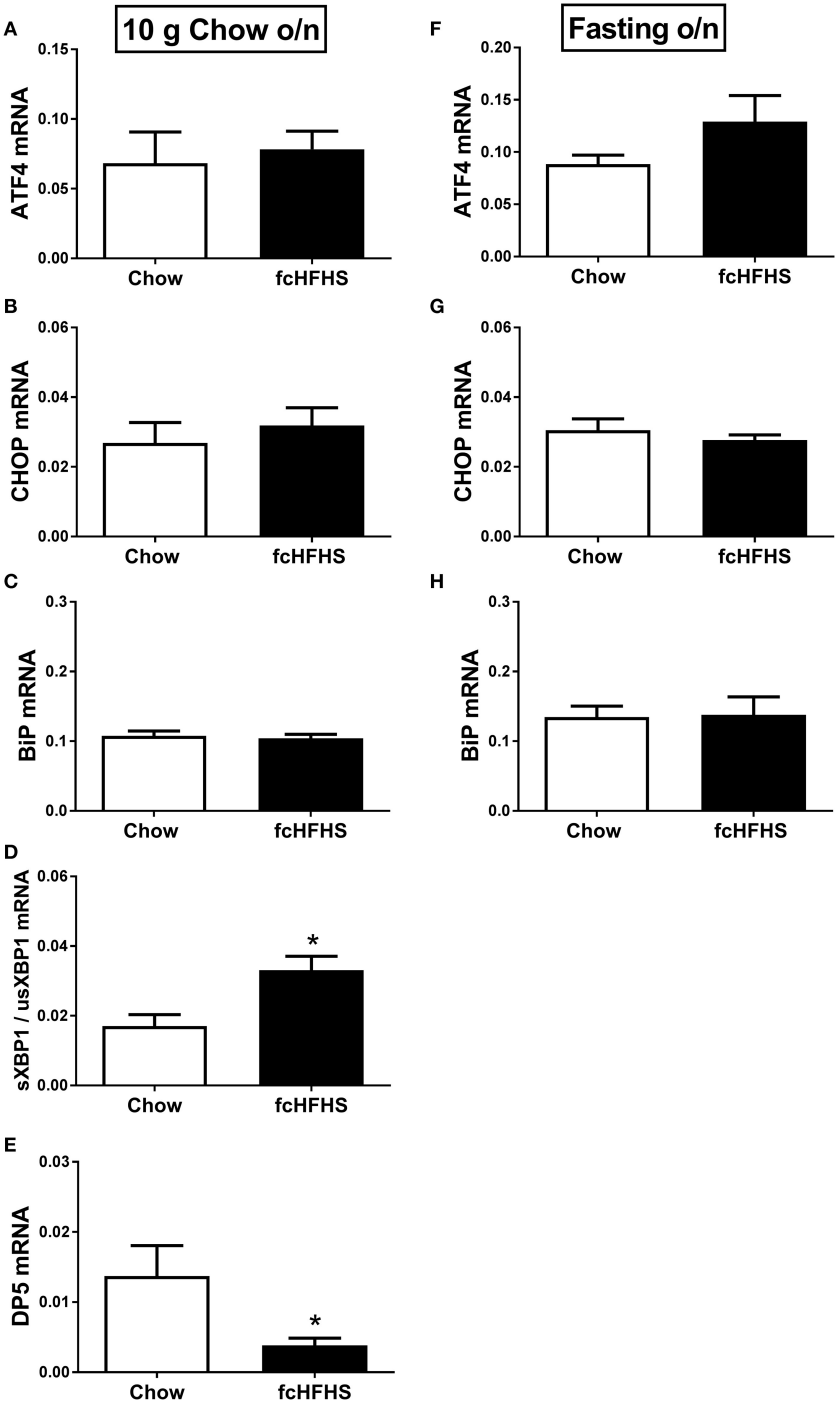
**The effect of overnight fat/sugar deprivation (10 g chow; left column) or fasting (right column) after 1 week fcHFHS diet or chow on the mRNA expression of hypothalamic (A,F)** ATF4, **(B,G)** CHOP, **(C,H)** BiP, **(D)** sXBP1/usXBP1, and **(E)** DP5. mRNA expression of specific genes was normalized to the geometric mean of three housekeeping genes. Significant differences between the fcHFHS and control groups: ^*^p < 0.05.

ΔBW, % WAT/BW and leptin were still increased in fcHFHS-fed rats compared to chow-fed rats when overnight deprived of fat and sugar (Table 3), but no changes were observed in hypothalamic ATF4, CHOP, and BiP mRNA expression between the groups (Figures 2A–C; left column). However, sXBP1/usXBP1 mRNA was higher and DP5 mRNA was lower in the fcHFHS-fed group compared to the chow controls (Figures 2D,E; left column).

No change in FasL mRNA expression was observed between the groups after removing fat and sugar overnight (not shown). Similarly, no differences in ATF4, CHOP, BiP, and FasL mRNA expression were observed between the groups after overnight fasting (Figure 2, right column). sXBP1/usXBP1 and DP5 mRNA expression were not detectable in the overnight fasted groups.

### Fat consumption leads the changes induced in stress gene markers

To determine to what extent fat or sugar contributed to the observed differences observed in hypothalamic mRNA, we divided the fcHFHS group according to fat and sugar intake using a median split forming either low or high fat consumers (LF or HF) or low or high sugar consumers (LS or HS). The median fat consumption was 10% out of total caloric intake. Rats that consumed <10% fat were assigned as LF consumers and those that consumed >10% as HF consumers. LF and HF consumers had similar BW, total caloric intake and chow intake, but fat consumption in HF group was significantly higher and sugar intake significantly lower compared to the LF group (Table 4). ATF4 mRNA expression tended to be higher (*p* = 0.07), in the hypothalamus of the HF consumers compared to the LF consumers, whereas sXBP1/usXBP1, DP5 and FasL mRNA expression was not different between the groups (data not shown). Similar analysis was performed for consumption of sugar over a week of fcHFHS exposure and median sugar consumption out of total caloric intake was 38%. No differences in ATF4, usXBP1, sXBP1/usXBP1, DP5, or FasL mRNA expression were observed between HS and LS consumers (data not shown).

**Table 4:** **Characteristics of low fat and high fat consumers over 1 week of fcHFHS diet**.

	**Total (Kcal)**	**Chow (Kcal)**	**Fat (Kcal)**	**Sugar (Kcal)**	**BW (g)**	**% FAT/BW**
HF	105 ± 2.3	50 ± 1.9	19 ± 1.40	36 ± 0.8	331 ± 1.8	3.26 ± 0.06
LF	98 ± 2.6	47 ± 3.0	6.9 ± 0.44^**^	49 ± 1.4^**^	331 ± 5.2	3.07 ± 0.08

Average Total, Chow, Fat, or Sugar caloric intake as well as body weight and % Fat/BW of animals over 1 week on fcHFHS diet. High fat (HF) and low fat (LF) animals were determined using median split. The median of fat intake was 10% out of total caloric intake. Significant differences between the HF and LF group:

** *p < 0.01*.

**Figure 3:**
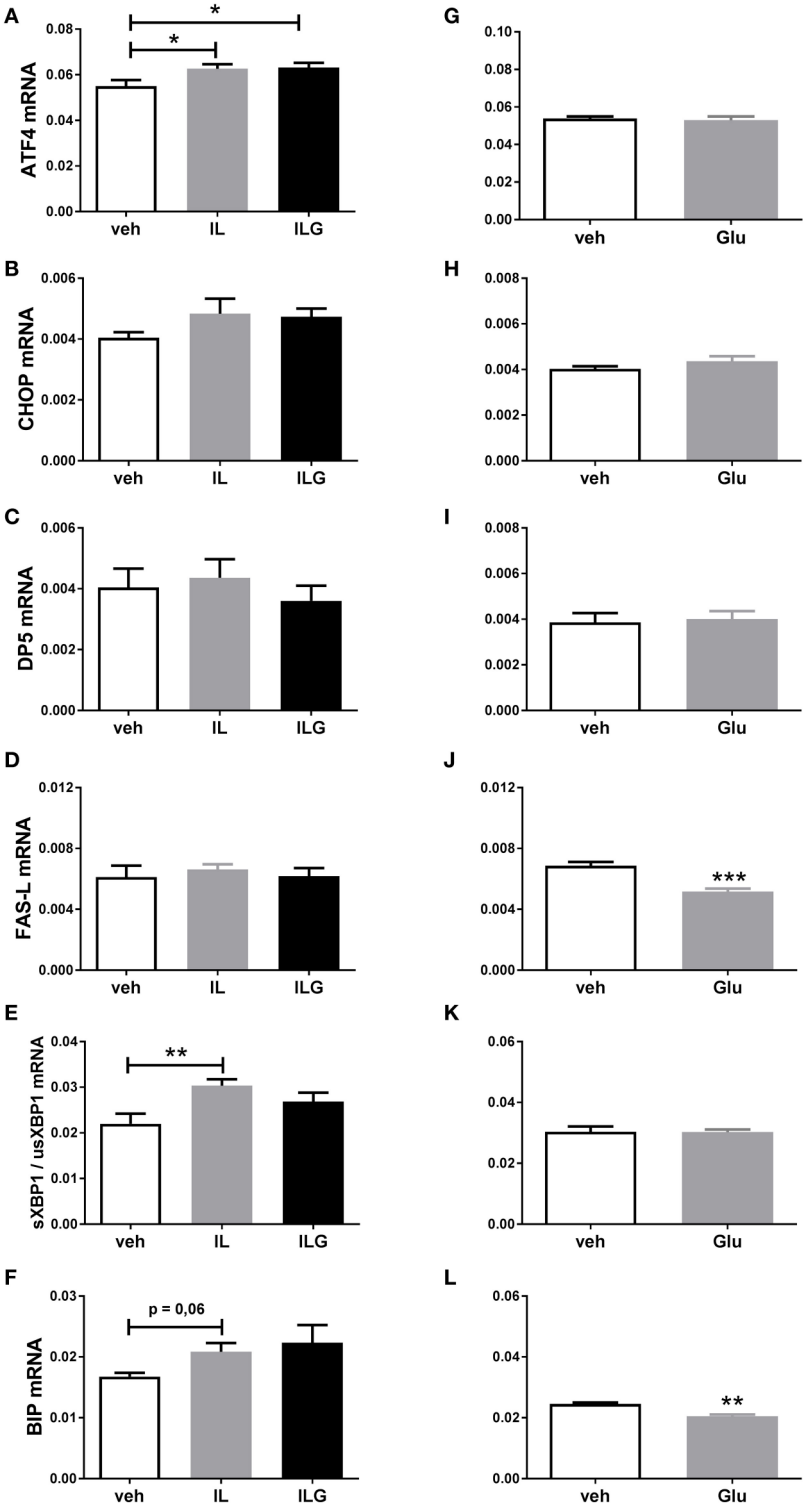
**The effect of central infusion of Intralipid (IL), Intralipid and Glucose (ILG) or Glucose on hypothalamic (A,G)** ATF4, **(B,H)** CHOP **(C)**, **(C,I)** DP5, **(D,J)** FasL, **(E,K)** sXBP1/usXBP1, **(F,L)** Bip **mRNA expression in chow rats**. mRNA expression of specific genes was normalized to the geometric mean of three housekeeping genes. Significant differences between groups: ^*^p < 0.05, ^**^p ≤ 0.01; ^***^p < 0.001.

To further investigate the direct effects of fat and sugar in the brain on hypothalamic ER stress markers, we infused IL, IL + glucose (ILG), glucose or saline (control) via the carotid artery directly to the brain of overnight fasted rats. IL and ILG increased hypothalamic ATF4 mRNA expression compared to saline but did not affect CHOP, DP5, and FasL mRNA (Figures 3A–D). In addition, IL infusion resulted in increased sXBP1/usXBP1 mRNA expression, and tended to increase BiP mRNA expression (Figures 3E,F). Glucose infusion did not have an effect on AFT4, sXBP1/usXBP1, and DP5 mRNA expression (Figures 3G–I,K), but reduced BiP and FasL mRNA levels compared to saline infusion (Figures 3J,L).

## Discussion

We showed that short term exposure to a fcHFHS diet, followed by mild food restriction overnight induces hypothalamic ER stress in rats as characterized by an increase in sXPB1/usXBP1 mRNA ratio in hypothalamus of fcHFHS fed rats compared to chow fed rats. Moreover, we showed that lipids, directly infused towards the brain of overnight fasted rats, are able to induce a similar response. As non-restricted *ad libitum* fcHFHS-diet fed or totally fasted rats do not show altered ratios, these data point to an interaction of lipid exposure to the brain and a negative energy balance in ER stress induction. In addition, we observed an increase in ATF4 mRNA when animals were *ad libitum* fed the fcHFHS diet which is abolished when rats are either provided with 10 g of chow overnight or totally fasted. In addition, high fat consumers on the fcHFHS diet have higher ATF4 mRNA, pointing to a direct role for fat intake in the increase in hypothalamic ATF4 mRNA. Indeed, ATF4 mRNA is also increased when animals are directly infused with lipids or lipids and glucose towards the brain, but not when glucose is infused alone.

An increase in sXPB1/usXBP1mRNA ratio implies activation of the IREα pathway (Yoshida et al., 2001; Calfon et al., 2002). The activation of IREα pathway due to HFD-feeding has been reported earlier. However, this was after long-term (8 or 20 weeks) exposure (Ozcan et al., 2009; Won et al., 2009). Here we showed for the first time that short term exposure to a fcHFHS diet is enough to induce activation of this pathway, but only under mild food restriction.

It is unclear why animals on a fcHFHS diet display this response only when food restricted while absent when animals are *ad libitum* fed overnight. One possibility might be that ER stress was induced via anabolic processes as illustrated by the observation that overnight fasting and subsequent refeeding increases sXBP1 mRNA and protein in the liver (Deng et al., 2013) and in the hypothalamus of mice (Williams et al., 2014). It is also possible that protective mechanisms play a role under *ad libitum* conditions. For example, we observed recently that 1 week fcHFHS diet increases beta- oxidation genes in the hypothalamus (Rijnsburger et al., 2016), and since enhanced beta-oxidation has been reported to counter palmitate-induced ER stress in *in vitro* models (McFadden et al., 2014), it is possible that we do not observe increased splicing of XPB1 under *ad libitum* feeding because of counter-active regulatory fatty acid oxidation. In line, we observed no changes in fatty acid oxidation genes after lipid infusion directly to the brain while it does induce XBP1 splicing (M. Rijnsburger, unpublished data). Further research is needed to determine the exact role of energy status and nutrient sensing on hypothalamic ER stress induction.

We observed a clear increase in hypothalamic ATF4 mRNA in rats on the fcHFHS diet while being *ad libitum* fed or when infused with intralipids via the carotic artery compared to vehicle infusions. However, we did not observe induction of downstream targets. It might be possible that increased ATF4 mRNA expression in our models is associated with other general stress related events within a cell i.e., amino-acid or glucose deprivation (Siu et al., 2002) or less protein availability (which has been shown in muscle to result in UPR activation; Deldicque et al., 2010). An additional effect of the fcHFHS diet is reduced chow—and thus protein—intake compared to chow controls, it is well possible that this might be the cause for the changes in ATF4 mRNA expression observed in our study. It is unclear at this point whether this increase in ATF4 mRNA will result in increased ATF4 protein. Interestingly, overexpression and inhibition of ATF4 in the hypothalamus induces hepatic insulin resistance and improves hepatic insulin sensitivity, respectively (Zhang et al., 2013). In addition, a recent study showed that ATF4 deletion in AGRP neurons of the hypothalamus specifically protects against high fat diet induced weight gain and insulin resistance (Deng et al., 2016). Together, this suggests an important role for hypothalamic ATF4 in the regulation of energy metabolism. Interestingly, we previously reported hepatic insulin resistance in rats on a fcHFHS diet (Diepenbroek et al., 2017), which could well be related to the observed increase in hypothalamic ATF4 mRNA.

Under severe ER stress, IREα activation can also lead to cell apoptosis via the JNK pathway (Todd et al., 2008) and to activation of apoptotic genes (Urano et al., 2000; Guan et al., 2006; Kim et al., 2006; Ma et al., 2007). We therefore measured DP5 and FasL mRNA, target genes of JNK pathway (Schenkel, 2004; Guan et al., 2006; Ma et al., 2007). However, 1 week fcHFHS diet did not result in activation of apoptotic gene expression. It could well be that this is due to the duration of the diet, as exposure to the diet for a longer period induces apoptosis accompanied by obesity and other metabolic disturbances (Ozcan et al., 2009; Won et al., 2009).

Interestingly a decrease in DP5 mRNA expression was observed in the fcHFHS group after overnight mild food restriction (10 g chow intake). That might be related to the increase in sXBP1 mRNA under the same conditions. Since both are regulated by IREα activation, the observed alterations suggest a shift from cell death related pathways to the induction of cell survival mechanisms. Like DP5, FasL mRNA can be also induced upon IREα/JNK activation during neuronal apoptosis (Le-Niculescu et al., 1999; Schenkel, 2004; Chen et al., 2015). However, no change was observed in FasL expression indicating that FasL does not play a major role in our experimental setting. In addition, a glucose infusion directly to the brain lowered FasL mRNA expression when compared to a vehicle control infusion, however what this means physiologically remains to be determined.

In summary, a fcHFHS diet and overnight fat and sugar availability affects the mRNA expression of hypothalamic ER stress related UPR markers. Overall, the UPR markers seemed to be a sensitive sensor of fatty acid availability as well as nutrient load. More studies are necessary to define the exact role of nutrients in induction of UPR intermediates that play a role in cellular metabolism and viability.

## Author contributions

EB, AB, and Sl designed experiments. EB, MR, LE, UU, and Sl performed experiments. EB, AB, and Sl prepared the manuscript. WS, MR, LE, and UU edited the manuscript. The entire study was supervised by AB and Sl.

### Conflict of interest statement

The authors declare that the research was conducted in the absence of any commercial or financial relationships that could be construed as a potential conflict of interest.
